# Be resistant to apoptosis: a host factor from gingival fibroblasts

**DOI:** 10.1038/cddis.2015.350

**Published:** 2015-12-03

**Authors:** R Cheng, T Hu, N A Bhowmick

**Affiliations:** 1 State Key Laboratory of Oral Diseases, Department of Preventive Dentistry, West China Hospital of Stomatology, Sichuan University, Chengdu, China; 2 Department of Medicine, Cedars-Sinai Medical Center, Los Angeles, CA, USA

Bacteria are the main cause of infectious diseases. Millions of bacteria colonized around gingival tissue can cause periodontal diseases.^1^ When dental plaque (bacteria) accumulates, the proportion of gram-negative and anaerobic bacteria increases for the release of endotoxin and bacterial debris (including lipopolysaccharide, LPS) into the gingivae. Then gingivitis, which is usually presented as swelling and bleeding in gingival tissue, may appear. A classic experiment has indicated healthy adults developed clinical signs of gingivitis without tooth cleaning in 2 or 3 weeks.^2^ But gingivitis is completely reversible in a week if oral hygiene is regained. If tooth cleaning is effective, dental plaque will be maintained in a relatively small amount and the gingivae remains comparably healthy.^3^

Gingivitis also represents a host–microbe homeostasis at the periodontal tissue, where the natural accumulation of microorganisms results in a limited and local host response without permanent damage to the host (mild inflammation).^4^ On the contrary, chronic periodontitis, an irreversible periodontal disease (severe inflammation), represents an uncontrolled host response. At present, periodontitis is classified as a disease of dysbiosis, where imbalanced host–microbe interaction is considered as a main reason for disease initiation and progression.^4^ The imbalanced host factors may greatly depend on the host's immune, inflammatory, and regenerative responses.^5^

So far the immune responses influencing host–microbe interaction are acknowledged. Neutrophils and regulatory T-cells (Tregs) are two pivotal immune cells in periodontitis. It has long been recognized that massive accumulation of neutrophils was one of the hallmarks of periodontitis. Diminished or excessive neutrophil function may cause early-onset periodontitis or chronic periodontitis.^6^ Tregs, a central negative regulator of host immune response, plays a role in the prevention of exaggerated responses to foreign harmful antigens. Tregs ensures a balanced immune response while localizes the damage to the environment where the response takes place.^4^ The protective role of Tregs has been confirmed, when disabling Tregs function resulted in deteriorated experimental periodontitis and decreases regulatory cytokines IL-10 and TGF-*β*.^7^

However, host response is more than host immune functions. The periodontal tissue, for example, periodontal ligament and gingivae have important roles in maintaining the structural integrity, in healing processes, and in pathological alterations.^8^ In our new study published in *Cell Death Discovery*, we describe that gingival fibroblasts can resist apoptosis in a period of time.^9^ This publication gives us a new insight into host–microbe homeostasis, that the reaction of gingival fibroblasts (target cells) to bacterial toxin may be a part of host response in periodontal diseases.

Gingival fibroblasts were stimulated by LPS *in vitro* to simulate acute inflammation (gingivitis). Reactive oxygen species (ROS) buildup, which represents oxidative stress in gingival fibroblast, was observed after 24 h. DNA damage was expected as a result of ROS accumulation. A time-dependent accumulation of DNA double-stranded breaks was evident after 48 h of LPS-induced oxidative stress. DNA damage may potentiate apoptosis or senescence as a factor of p53 status. As expected, p53 had increased as a response to acute LPS exposure. Then the expectation was the apoptotic death of DNA damaged cells. However, apoptosis was not detected after 24 and 48 h of LPS exposure.

In our *Cell Death Discovery* publication, possible anti-apoptotic mechanisms were tested. Not surprisingly, pro-apoptotic proteins, p53, phosphorylated JNK, and phosphorylated p38 were activated (Figure 1). But, the elevation of anti-apoptotic proteins, BCL-1, phosphorylated ERK1/2, and Survivin likely countered apoptotic effects. It is also worth noting the pro-survival kinase, phosphorylated AKT was decreased. However, in contrast to its usual ability, decreased active AKT could inhibit ROS-mediated apoptosis.^10^ As a result, the anti-apoptotic effects and pro-apoptotic effects seemed to be balanced. Cell apoptosis was not induced by LPS in acute exposures. Apoptotic resistance of gingival fibroblasts is consistent with the observed rapid recover from gingivitis upon proper oral hygiene. Importantly, apoptotic gingival fibroblasts were unavoidable in the periodontitis models involving chronic LPS exposure as well as in patients, supporting the irreversibility of the disease.

The alternative consequences of gingival fibroblast DNA oxidation and damage include senescence and autophagy. Both of them were observed in this study. Fibroblast DNA damage–associated senescence may have been initiated even in the gingivitis stage. Based on the literature describing the paracrine proliferative secretome expressed by senescent fibroblasts, it would suggest that the senescing fibroblast population could potentiate the expansion of the other neighboring fibroblasts.^11^

The publication illustrates a new insight that gingivitis could be curable if the bacterial insult can be removed in a timely manner. The self-defense ability may also help to keep gingivae ‘healthy' during the interval of tooth cleaning. Contrary to gingivitis, chronic periodontitis induced cell damage *in vivo*, including DNA damage and cell apoptosis. It adds evidence that periodontitis leads to cell and tissue damage. The paper confirms early intervention to periodontal diseases is of the most importance. If some measures could be taken to prolong the self-recovery time, the periodontal tissue might be more resistant to bacterial toxin.

## Figures and Tables

**Figure 1:**
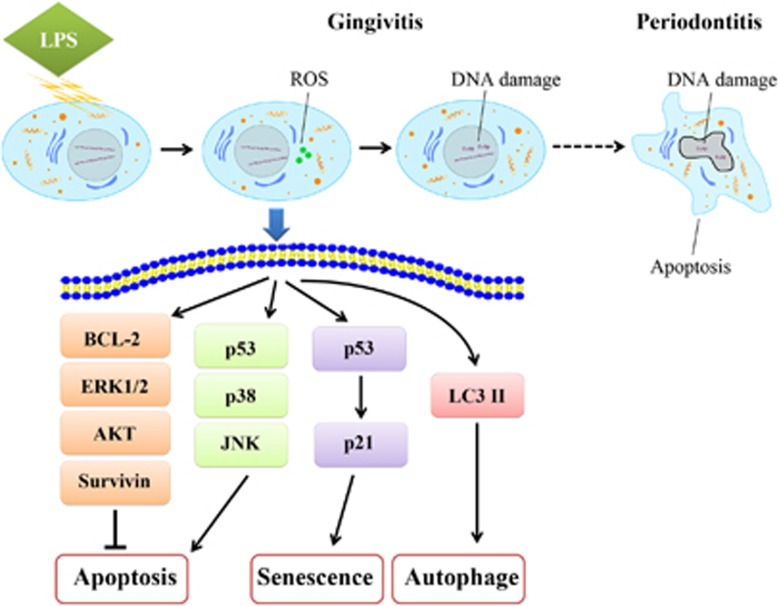
Mechanisms of gingival fibroblasts in cell apoptosis resistance. LPS-induced ROS accumulation in acute inflammation. DNA damage appeared as time went on. Finally, apoptosis was detectable in chronic periodontitis. Short after stimulation, LPS increased phosphorylated ERK1/2, phosphorylated p38, phosphorylated JNK, p53, BCL-2, and Survivin in a dose-dependent manner. Simultaneously, phosphorylated AKT decreases in a dose-dependent manner. As a result, the anti-apoptotic effects and pro-apoptotic effects were balanced. The expression of p53 and p21, the ratio of LC3 ІI/LC3 I were increased by LPS, indicating senescence and autophagy were involved, respectively
